# Varied roles of Pb in transition-metal Pb*M*O_3_ perovskites (*M* = Ti, V, Cr, Mn, Fe, Ni, Ru)

**DOI:** 10.1088/1468-6996/16/3/036003

**Published:** 2015-06-04

**Authors:** John B Goodenough, Jianshi Zhou

**Affiliations:** Materials Science and Engineering Program and Texas Materials Institute, University of Texas at Austin, Austin, TX 78712, USA

**Keywords:** perovskites, lone pair electrons, crystal structure

## Abstract

Different structural chemistries resulting from the Pb^2+^ lone-pair electrons in the Pb*M*O_3_ perovskites are reviewed. The Pb^2+^ lone-pair electrons enhance the ferroelectric transition temperature in PbTiO_3_, stabilize vanadyl formation in PbVO_3_, and induce a disproportionation reaction of Cr^IV^ in PbCrO_3_. A Pb^2+^ + Ni^IV^ = Pb^4+^ + Ni^II^ reaction in PbNiO_3_ stabilizes the LiNbO_3_ structure at ambient pressure, but an *A*-site Pb^4+^ in an orthorhombic perovskite PbNiO_3_ is stabilized at modest pressures at room temperature. In PbMnO_3_, a ferroelectric displacement due to the lone pair electron effect is minimized by the spin–spin exchange interaction and the strong octahedral site preference of the Mn^IV/III^ cation. PbRuO_3_ is converted under pressure from the defective pyrochlore to the orthorhombic (*Pbnm*) perovskite structure where Pb–Ru interactions via a common O −2p orbital stabilize at low temperature a metallic *Imma* phase at ambient pressure. Above *P*_c_ 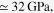 a covalent Pb–Ru bond is formed by Pb^2+^ + Ru^IV^ = Pb^4+^ + Ru^II^ electron sharing.

## 1. Introduction

The Pb*M*O_3_ perovskites manifest an interplay between the Pb–6s,6p lone-pair hybrids and the transition-metal *M*O_3_ perovskite framework. The classic case of ferroelectricity in PbTiO_3_ has been well-discussed and understood in the literature. The application of high-pressure synthesis in recent years has made available Pb*M*O_3_ with *M* covering almost all 3d transition metals and revived interest in the study of PbRuO_3_. The relevant features of this *M*O_3_ framework with which the Pb^2+^ lone pairs interact are reviewed in the discussion of the properties of each Pb*M*O_3_ compound.

The geometric perovskite tolerance factor

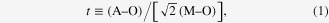
where (*A*–O) and (*M*–O) are the equilibrium bond lengths, is a useful parameter. Compounds *AM*O_3_ with a *t* < 1 can be stabilized in the perovskite structure by a cooperative rotation of the MO_6/2_ octahedra. The versatility of the *M*O_3_ framework has provided a wealth of information on the properties of the d electrons in the transition-metal *AM*O_3_ perovskites [1]. However, with a *t* > 1, the (*M*–O) bonds are either stretched beyond their equilibrium length or the structure transforms to a hexagonal polytype [2]. Where the (*A*–O) bond is more compressible than the (*M*–O) bond, high pressure lowers the effective tolerance factor to stabilize the cubic perovskite structure, and the cubic perovskite is commonly retained at ambient pressure and temperature. Most of the Pb*M*O_3_ perovskites are synthesized under high pressure at high temperature.

In a cubic *AM*O_3_ perovskite, the oxide-ion orbitals that *π*-bond with the *M*-cation d orbitals also σ-bond with the larger *A* cation in the body center of the *M*O_3_ framework. Competition for covalent bonding with those O–2p electrons means that the stronger is the covalent component of the *A*–O bond, the weaker is the covalent component of the *M*–O p_*π*_ bonding and therefore the weaker the interatomic *M*–O–*M* interactions via the O–2p_*π*_ bonding of the *M*O_3_ framework. This competition is particularly significant for Pb*M*O_3_ perovskites where the d electrons of the *M*O_3_ framework are at the crossover from localized to itinerant behavior as a result of the *M*–O–*M* interactions; the equilibrium (*M*–O) bond length is larger for localized than for itinerant d electrons.

The large mass of the Pb atom separates the energies of the 6s and 6p electrons; the 6s electrons are more stable than the 6p electrons because the 6s wave functions have a finite density at the nucleus whereas the 6p wave functions have a node at the nucleus. The larger the mass of the atom, the greater the difference in the screening of the outer s and p electrons from the nuclear charge by the core electrons. As a result, two cation valences are stable: Pb^2+^: 6s^2^6p^0^ and Pb^4+^:6s^0^6p^0^. Hybridization of the 6s and 6p orbitals creates an electron density with its center displaced from the nucleus; and in an oxide, the energy cost to form a 6s,6p hybrid can be more than compensated by the gain in bond energy by the covalent component of the Pb–O bond with the empty hybrid orbital on the opposite side of the nucleus. This hybridization is the origin of the lone-pair phenomenon of a Pb^2+^ ion in an oxide. However, in a Pb*M*O_3_ perovskite, the increased Pb–O covalent bonding competes with *π*-bonding d-orbital covalent bonding, which introduces an interplay between the lone pair on a Pb^2+^ ion and orbital ordering or the strength of the *M*–O–*M* interactions in the *M*O_3_ framework. All data of crystal structure and physical properties of Pb*M*O_3_ (*M* = Ti, V, Cr, Mn, Fe, Ni, Ru) reviewed in this article are listed in table 1

**Table 1.** Crystal structure and lattice parameters at room temperature and physical properties of Pb*M*O_3_.

	PbTiO_3_	PbVO_3_	PbCrO_3_	PbMnO_3_	PbFeO_3_	PbNiO_3_	PbRuO_3_
Symmetry at room temperature	*P*4*mm*	*P*4*mm*	*Pm*-3*m*	*P*4/*mmm*	Orthorhombic	*Pbnm*	*Pbnm*
Lattice parameters (Å)	*a* = 3.9009 [3]	*a* = 3.800 05 [5]	*a* = 4.0054 [7]	*a* = 3.8561 [9]	*a* = 3.908 [10]	*a* = 5.358 27 [11]	*a* = 5.614 30 [12]
*c* = 4.1526	*c* = 4.6703		*c* = 3.9209	*b* = 3.948	*b* = 5.463 25	*b* = 5.563 14
				*c* = 3.866	*c* = 7.707 41	*c* = 7.864 68
Physical properties	Ferroelectric *T*_c_ = 764 K [4]	Ferroelectric *T*_c_ = ?	Antiferromagnetic	Antiferromagnetic	?	Antiferromagnetic	Paramagnetic
		Antiferromagnetic *T*_N_ = 180 K[6]	*T*_N_ = 245 K [8] CD [7]	*T*_N_ ∼ 40 K [9]		*T*_N_ ≈ 225 K[11]	metal [13,14]

CD: charge disproportionation.

## 2. Data and discussion

### 2.1. PbTiO_3_

A *t* > 1 in BaTiO_3_ stretches the Ti–O bond lengths to make the symmetric O–Ti–O bonds along the 〈100〉 axes unstable relative to a cooperative, ferroelectric displacement of the Ti^IV^ towards one of its two neighbors: first along one principal axis, then along two, and finally along all three axes as the temperature is lowered [4]. With a room-temperature *t* 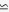 1, SrTiO_3_ remains cubic to below room temperature, undergoing a cooperative rotation of the TiO_6/2_ octahedra about a principal axis below 110 K to increase the Sr–O bond energy [15]. Since the ground state is nearly degenerate with a ferroelectric phase, an ^18^O isotope substitution induces a soft-mode phase transition [16]. Although the size of the Pb^2+^ ion (1.63 Å) is comparable to that of the Sr^2+^ ion (1.58 Å) (tabulated value for XII coordination), nevertheless the difference is large enough to create a *t* > 1 that induces a ferroelectric displacement of the Pb^2+^ by a hybridization of the Pb–6s,6p orbitals. As a results, PbTiO_3_ undergoes a cooperative ferroelectric displacement of the Ti^IV^ along a single principal axis at a Curie temperature *T*_c_ = 764 K [17, 18], significantly higher than *T*_c_ = 393 K of BaTiO_3_ [19]. PbTiO_3_ has a room-temperature axial ratio *c/a* = 1.064 and exhibits a negative thermal expansion to *T*_c_ because the cation displa_c_ements that enhance the bonding also increase the volume [17, 18]. In PbTiO_3_, ordering of the lone pair on the Pb^2+^ ions along a principal axis stretches the O–Ti–O bond lengths in this direction to induce the ferroelectric Ti^IV^ displacement. The *c*-axis O–2p_*π*_ orbitals are not impacted by the high-temperature transition of the Pb^2+^ lone-pair hybrid orbitals; only those in the basal plane. The interplay between the lone-pair ordering on the Pb^2+^ and the ferroelectric Ti^IV^ displacements of the TiO_3_ framework yields a high ordering temperature and restricts the Ti^IV^ displacement to a single axis. The ferroelectric displacement in PbTiO_3_ has been fully justified by the density functional theory (DFT); readers are referred to [20] for more detailed information.

### 2.2. PbVO_3_

An octahedral-site V^IV^ has a single d electron in a threefold-degenerate manifold of *π*-bonding orbitals. In the perovskites CaVO_3_ and SrVO_3_, the V–O–V interactions are strong enough to create an itinerant-electron band 1/6-filled; these perovskites are metallic [21]. On the other hand, the single d electron per Ti^III^ in the RTiO_3_ perovskites (R = Y or a rare earth) are localized [22]. The smaller Δ*E* to transfer an O–2p electron to an empty *π*-bonding *t*_2_ orbital of V^IV^ enhances the covalent contribution to the V–O bond, thereby increasing the interatomic V–O–V interactions.

Whereas the orbital degeneracy of a localized-electron manifold is removed by a cooperative Jahn–Teller orbital ordering, as is illustrated in the RTiO_3_ perovskites, the itinerant-electron orbital degeneracy of a V^IV^ may be removed by formation of the vanadyl ion (V = O)^2+^ in which the d electron is ordered into a plane perpendicular to the V = O bond axis. The vanadyl formation places the V^IV^ in a square-pyramidal site with five rather than six near-neighbor oxide ions.

This situation is well-illustrated by VO_2_, which crystallizes in the rutile structure, but undergoes a first-order metal-insulator transition below 340 K. The low-temperature monoclinic phase is characterized by a cooperative shift of the V^IV^ ions perpendicular to the *c*-axis to form short V = O bonds with an ordering of the single d electron per V^IV^ into a narrow *c*-axis band that is split in two by a charge-density wave of V–V dimers containing V–V bonding across shared site edges [1].

**Figure 1.**
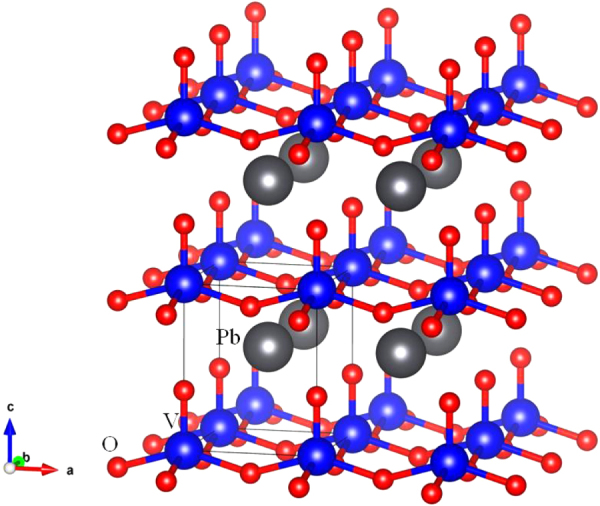
Crystal structure of perovskite PbVO_3_ (based on the CIF data from [5]).

With this background, we now look at the structure of PbVO_3_ prepared under high pressure at high temperature [5]. The lattice instabilities associated with the lone-pair ordering on the Pb^2+^ together with lattice instabilities associated with orbital degeneracies stabilize competitive phases in attempts at ambient-pressure synthesis. Nevertheless, under high pressure at high temperature, the PbVO_3_ phase is preferred and is retained on removal of the pressure at room temperature. Like PbTiO_3_, the high-pressure PbVO_3_ phase is tetragonal under ambient conditions with the V atoms displaced from symmetric O–V–O bonding along the *c*-axis; but where PbTiO_3_ has a room-temperature axial ratio *c/a* = 1.062, PbVO_3_ has a much larger *c/a* = 1.229 as a result of formation of the vanadyl (V = O)^2+^ ion [5]. The tetragonal PbVO_3_ structure is shown in figure 1. The single d electron per V^IV^ is localized and ordered into the *xy* orbital in the basal plane; the in-plane interatomic V–O–V interactions between the localized-electron spins give a broad maximum in the paramagnetic susceptibility near 200 K [6] typical of 2D antiferromagnetic interactions [24].

**Figure 2.**
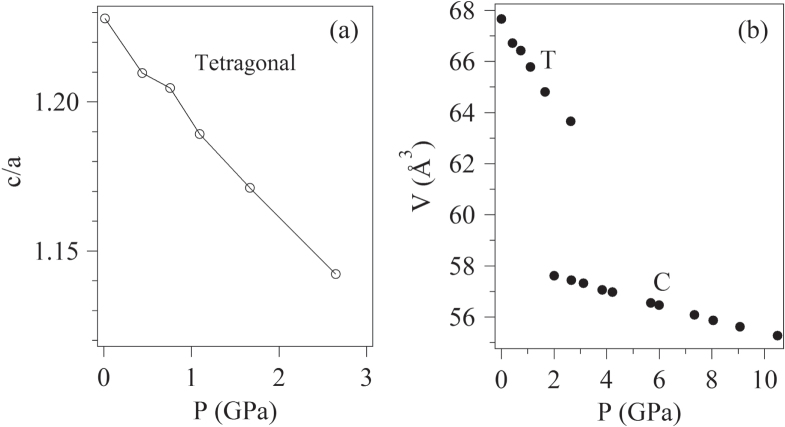
Lattice parameters as a function of pressure for perovskite PbVO_3_, data are after [26].

Since the high-pressure synthesis at high temperature favors the PbVO_3_ phase, it provides a means of obtaining a nearly pure PbVO_3_ phase. Once this phase is quenched to ambient pressure and room-temperature, a natural next step is to investigate what happens to this tetragonal phase when it is subjected to high pressure at room temperature. High pressure can be expected to change the V^IV^ coordination from square-pyramidal to octahedral by a reduction of both the *c*-axis V displacements from an asymmetric to a symmetric bond and by suppression of lone-pair 6s, 6p hybridization until a cubic PbVO_3_ perovskite structure is attained. It took 10 years for this investigation to be performed, and then the motivation was a search for a new high-temperature superconductor. However, this investigation proves to be of fundamental importance for our understanding of the transition in a single-valent oxide from localized to itinerant d-electron behavior. In the Mott–Hubbard scenario, the dynamics of electron lattice interactions is neglected; orbital degeneracies are removed by the itinerant-electron dispersion curve of width *W*, and the electron–electron correlation energy *U* associated with excitations of electrons to atomic states of lower positive valence is able to open a gap in the density of one-electron states where a *U* > *W* is realized with a reduced bandwidth *W*. This scenario is the basis of the DFT + *U* calculations in which *U* appears as an adjustable parameter. The alternative view for a mixed-valent system is to invoke strong electron–lattice interactions that trap electrons in *M*-cation sites with (*M*–O) bond lengths appropriate to the *M*-cation valence. Alternatively, it has been argued from the Virial Theorem for central-force fields [25] that the equilibrium (*M*–O) bond length for itinerant electrons (Mott–Hubbard scenario) should be smaller than that for localized electrons and, therefore, that there can be a first-order bond contraction at a transition from localized to itinerant electrons in a single-valent system like PbVO_3_. The DFT + *U* calculations work well for a single-valent *M*O_3_ configuration with itinerant or localized d electrons because a periodic potential is preserved in a single-valent compound. Figure 2(a) shows the anticipated reduction in the axial ratio *c/a* of the tetragonal PbVO_3_ phase under pressure at room temperature over the pressure range 0 < *P* ≤ 2.5 GPa and figure 2(b) shows a first-order phase change between the tetragonal and cubic phase in the interval 1.16 < *P* < 3.82 GPa. However, despite a drop in resistivity by 5 orders of magnitude across the transition, this itinerant-electron cubic phase is still semiconductive between 2 and 300 K up to 11.3 GPa, indicating a *U* > *W*. Since cubic SrVO_3_ is metallic, the data indicate that the Pb^2+^ ion is narrowing the *π*∗ bandwidth compared to its width in SrVO_3_. The availability of the Pb–6p^0^ orbitals for covalent bonding with the O–2p orbitals in competition with the V–O2p_*π*_ bond covalence reduces the interatomic V–O–V interactions in cubic PbVO_3_ relative to its value in cubic SrVO_3_; this reduction appears to render a *W* < *U* in PbVO_3_, but there is no evidence for either polaronic conduction in cubic PbVO_3_ or localized-electron orbital order. On removal of the high pressure, the cubic perovskite PbVO_3_ reverts back to the tetragonal phase in the interval 1.11 < *P* < 2.02 GPa, which has prevented the measurement of the magnetic properties of the strongly correlated itinerant-electron phase with *U* > *W*. Of particular interest would be the extent to which the itinerant-electron dispersion width *W* suppresses the intraatomic orbital angular momentum and spin relative to that for a localized-electron phase. The RTiO_3_ system contains a single localized *t*_*2*_ electron per Ti^III^ ion for comparison [22].

### 2.3. PbCrO_3_

Octahedral-site Cr^IV^ has two d electrons per Cr^IV^, which prevents removal of the d*-*state degeneracy by the formation of asymmetric O–Cr–O bonds; therefore, a *c*-axis ordering of the Pb^2+^ lone pairs can only be stabilized by a single-atom removal of a localized-electron orbital degeneracy on the (CrO_3_)^2−^ framework. The intra-atomic spin–spin interactions responsible for Hund’s highest multiplicity rule on a free Cr^IV^ ion can be expected to narrow the bandwidth *W* of a cubic (CrO_3_)^2−^ framework and to enlarge the correlation energy *U* to create a *U* > *W*; but a *π*∗ band 1/3-filled would be at the crossover from antiferromagnetic to ferromagnetic spin ordering, which can be unstable relative to a charge-disproportionation as is found in metallic Mn [27]. Nature resolves this problem in ferromagnetic CrO_2_ by providing a *c*-axis band as well as a *π*∗ band; the *π*∗ band is ¼-filled, and the remaining electrons are ordered into the *c*-axis band as localized electrons. The localized-electron spins are aligned parallel to the spins of a ferromagnetic *π*∗ band ¼-filled [27]. In a cubic- perovskite (CrO_3_)^2−^ framework, this option is not available. A remaining option for removal of the orbital degeneracy at an octahedral-site Cr^IV^ is a charge disproportionation (CD).

A CD of octahedral-site Cr^IV^ is illustrated by the spinel compound Li[MnCr]O_4_ [28]. As Li is removed, the Cr^III^ are progressively transformed to Cr^IV^ and the Li vacancies create empty tetrahedral sites in the interstitial space that do not share a face with a neighboring Li^+^ ion. These tetrahedral sites can accept a Cr^VI^ cation, which triggers the CD reaction 3Cr^IV^ = Cr^VI^ + 2Cr^III^. In a PbCrO_3_ perovskite, lone-pair hybridizations on the Pb^2+^ can perturb the (CrO_3_)^2−^ framework so as to allow a CD reaction within the framework. The *t*^*3*^ configurations on the Cr^III^ have a localized spin *S* = 3/2.

A cubic PbCrO_3_ perovskite phase was first stabilized under high pressure at high temperature by Roth and DeVries in 1967 [29, 30], but the structure exhibits several unusual properties. First, its lattice constant *a* = 4.00 Å is significantly larger than that expected based on the equilibrium (Pb–O) bond length for a Pb^2+^ ion. Second, it exhibits a Type-G antiferromagnetic order (all nearest-neighbor spins antiparallel) with a *T*_N_ = 240 K [29], nearly three times higher than the *T*_N_ = 90 K of CaCrO_3_; CaCrO_3_ has localized Cr^IV^ d electrons (its tolerance factor is *t* < 1) that are ordered to give Type-C antiferromagnetic order (ferromagnetic *c*-axis interactions) [31]. PbCrO_3_ also has a much higher resistivity and energy gap than are found in CaCrO_3_ and SrCrO_3_ [31]. In addition, PbCrO_3_ has a highly irregular peak profile in the powder x-ray diffraction pattern [32] and an unusually high thermal factor at the Cr site characteristic of a static disorder of the Cr atoms. By allowing the Cr atoms to occupy the general Wickoff position 8g (*x*,*x*,*x*) with *x* = 0.035, the overall intensity and peak profile matched well with the structural refinements. Although neutron diffraction does not show evidence of long-range order of the Cr displacements, the refinement of the oxygen positions shows a thermal parameter 10 times larger than is found in other Cr^IV^-containing perovskites: [33] *viz*. 2 Å^3^ versus 0.2 and 0.44 Å^3^. These observations are important experimental clues in support of a CD model, and a DFT + *U* calculation gave an independent prediction of the CD model [7].

**Figure 3.**
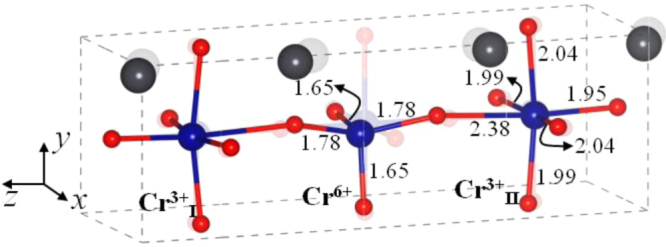
The optimized CD model structure with bond length in Å together with the ideal cubic structure shown as faded structure at the background; the data are from [7].

By allowing the cubic phase of PbCrO_3_ to expand its volume at room temperature by 10% on removing the high pressure, the calculation showed the volume expansion leads to significant atomic displacements from the ideal cubic positions. Two CD reactions were shown to be close in energy: 3^+^ + 3^+^ + 6^+^ and 3^+^ + 5^+^. The optimized model structure with the 3^+^ + 3^+^ + 6^+^ configuration is shown in figure 3. Pb atoms are displaced substantially towards Cr^III^, which are displaced slightly from the ideal cubic positions. The Cr^III^ and Cr^VI^ are coordinated, respectively, by six and by four oxygen nearest neighbors. In the distorted tetrahedron, there are two short Cr=O bonds and two relatively long Cr–O bonds. The two bond lengths, 1.65 and 1.78 Å are comparable to the 1.59 and 1.78 Å for the calculated Cr=O and Cr–O bonds in a tetrahedral CrO_2_(OH)_2_ cluster in the gas phase. The oxygen bridging to a neighboring Cr^III^ moves towards the Cr^VI^ to give a long 2.40 Å Cr^III^–O bond length while the other five Cr–O bonds of Cr^III^ are 2.00 ± 0.05 Å, close to a typical Cr^III^–O bond length of 2.0 Å in the RCrO_3_ perovskites [34]. The oxygen bridging from the Cr^III^ to the Pb^2+^ form Pb–O bond lengths of 2.43–2.56 Å, substantially shorter than the 2.85 Å estimated for the ideal cubic phase. These shorter Pb–O bonds indicate the Pb–6s,6p hybridization plays an important role in stabilizing the CD of the (CrO_3_)^2−^ framework.

**Figure 4.**
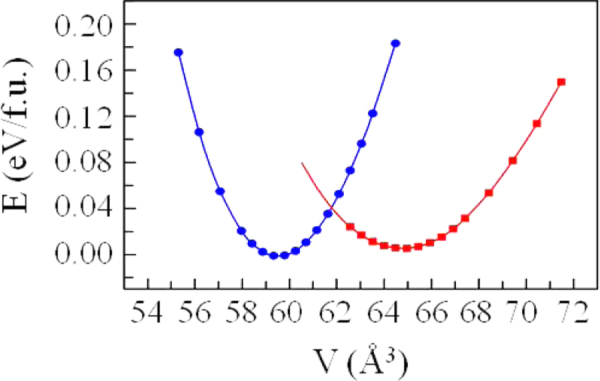
The relative energies (*E*) per formula unit (f.u.) for the ideal cubic perovskite (blue) and the CD (red) phases of PbCrO_3_ as a function of cell volume (*V*); the data are from [7].

Figure 4 shows the calculated relative energies *E* per formula unit for the ideal cubic perovskite (blue) and the CD (red) phases of PbCrO_3_ as a function of the cell volume *V*. The application of hydrostatic pressure at room temperature should suppress the CD model in favor of the ideal cubic model. Indeed, with a sample synthesized with laser heating under the pressure of a diamond anvil cell, an earlier report [35] signaled a large (9.8%) volume and resistivity collapse in a reversible cubic-cubic first-order transition at a *P*_c_ = 1.4 ± 0.2 GPa at room temperature. The abrupt transition was found at a *P*_c_ near 3 GPa in a sample synthesized with a large-volume press [7]. In this latter experiment, the large thermal parameters of the diffraction profiles were also found to drop precipitously in the high-pressure phase. The resistivity drops by over two orders of magnitude at *P*_c_; the conductivity of the high-pressure polycrystalline phase was characteristic of a narrow-band conductor with grain-boundary scattering. The magnetic properties of the high-pressure phase have not been determined because this phase is not retained on removal of the pressure.

### 2.4. Solid solutions

Arevalo-Lopez and Alario-Franco [36] have investigated the three solid-solution systems PbTi_1-*x*_V_*x*_O_3_, PbTi_1-*x*_Cr_*x*_O_3,_ PbV_1−*x*_Cr_*x*_O_3._ The system PbTi_1−*x*_V_*x*_O_3_ remains tetragonal for all *x* with a continuous increase with *x* of the *c* axis and volume. The tetragonal to cubic transition decreases from *T*_c_ = 763 K for *x* = 0–730 K for *x* = 0.2, but for *x* ≥ 0.3, the samples decompose on heating at ambient pressure. The authors suggest there is a percolation threshold for the PbVO_3_ phase near *x* = 0.3. Similarly, a percolation threshold for the PbCrO_3_ appears near *x* = 0.3 in PbTi_1−*x*_Cr_*x*_O_3_ and PbV_1−*x*_Cr_*x*_O_3,_ a tetragonal/cubic two-phase region appearing at *x* = 0.3 in the former and an abrupt tetragonal to cubic transition occurring in the interval 0.3 < *x* < 0.4 in PbV_1−*x*_Cr_*x*_O_3_. The Pb^2+^ displacements become disordered at the percolation threshold in the two Cr substituted systems.

### 2.5. PbMnO_3_

The octahedral-site Mn^IV^ ions of stoichiometric PbMnO_3_ should have a non-degenerate *t*^*3*^ manifold with a localized-electron spin *S* = 3/2 if there is no overlap of the Mn^IV^/Mn^III^ redox energy by the Pb–6s states. With no orbital degeneracy to be removed, the (MnO_3_)^2−^ framework of a cubic PbMnO_3_ can be expected to resist deformation of the cubic framework by ordering of a lone-pair on the Pb^2+^ ions. Except for a reduction of the Mn–O–Mn antiferromagnetic interactions compared to SrMnO_3_ by a larger competition of the O–2p orbitals for covalent bonding to the Pb^2+^–6p orbitals, the behavior of PbMnO_3_ might be similar to that of SrMnO_3_if the *t* > 1 does not stabilize a hybridization of the Pb^2+^−6s,6p electrons.

Both SrMnO_3_ and PbMnO_3_ have a tolerance factor *t* > 1, and both form a perovskite polytype. Under pressure at high temperature, both compounds crystallize in the 6H polytype in which the AO_3_ (111) planes of a cubic phase are stacked cubic, cubic, hexagonal along the *c*-axis. Application of higher pressure at high temperature normally converts 6H polytypes to the cubic 3C perovskite structure [2]. Oka *et al* [9] converted the 6H PbMnO_3_ polytype to the cubic perovskite structure by treating oxygen-stoichiometric 6H PbMnO_3_ under 15 GPa at 1000 °C. Unfortunately the cubic phase obtained was not oxygen-stoichiometric; iodometric titration showed a composition PbMnO_2_._94(1)_. SrMnO_3_ is notoriously sensitive to loss of oxygen [37], which stabilizes a mixed-valent phase by the introduction of Mn^3+^; the Mn^3+^ introduces a ferromagnetic component into the MnO_3_ framework. The small tetragonal (*c/a* = 1.017) distortion of their 3C PbMnO_3−*δ*_ and the reduction of the *T*_N_ to 20 K compared to 233 K for oxygen-stoichiometric 3C SrMnO_3_ [37] cannot be properly interpreted in terms of the relative properties of the Pb^2+^ and Sr^2+^ ions. As a matter of fact, refinement of the synchrotron x-ray diffraction pattern cannot distinguish the polar structure *P*4*mm* versus the non-polar structure *P*4*/mmm* of the 3C phase. A DFT calculation [38] indicates that the *P*4*mm* phase has a slightly lower energy of the ground state than that in the *P*4*/mmm* phase. However, the calculation also predicts the *P*4*mm* phase is metallic. It remains to be clarified whether the lone-pair electrons would induce a ferroelectric displacement in oxygen stoichiometric PbMnO_3_.

### 2.6. PbFeO_3_

Tsuchiya *et al* [10] have reported the synthesis of PbFeO_3_ under high pressure. In comparison with other Pb*M*O_3_ perovskites, the study of PbFeO_3_ is incomplete. The report just shows that the x-ray diffraction pattern of the high-pressure product can be indexed by an orthorhombic perovskite cell, indicating a reduction of the tolerance factor to a *t* < 1 and therefore a transfer of electrons from Pb^2+^ to Fe^IV^. The x-ray photoemission spectra indicate that the valance of iron in PbFeO_3_ is close to that of the Fe^III^ in LaFeO_3_. The Fe^IV^/Fe^III^ couple has an energy that favors the reaction Pb^2+^ + 2Fe^IV^ = Pb^4+^ +2Fe^III^, but Fe^III^ is stable in the presence of Pb^2+^. This situation is consistent with the observation of Fe^III^, but it leaves the valence state of Pb ambiguous: Pb^2+^ + Pb^4+^ versus Pb^3+^−6s^1^.

### 2.7. PbNiO_3_

**Figure 5.**
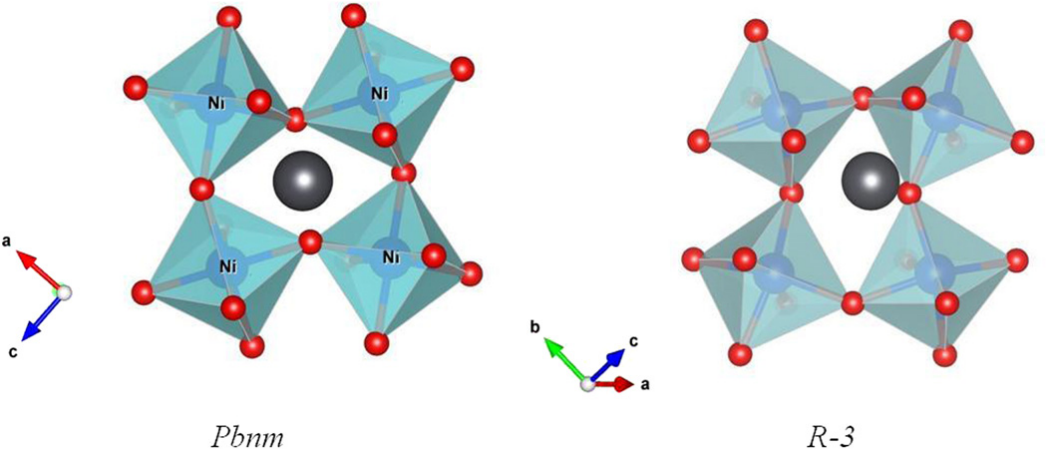
Two structural models of PbNiO_3_.

The Ni^IV^/Ni^III^ and Ni^III^/Ni^II^ redox energies are at the top of the O–2p bands [39], and removal of Li from LiNiO_2_ results in loss of O_2_ as nearly all the Li is removed [40]. It is easier to synthesize PbO_2_ than NiO_2_, which favors the reaction Pb^2+^ + Ni^IV^ → Pb^4+^ + Ni^II^ in PbNiO_3_. Although Pb^4+^ is stable in octahedral coordination, Inagauma *et al* [11] have synthesized an orthorhombic (tolerance factor *t* < 1) perovskite PbNiO_3_ at 3 GPa and 800°C; the Pb^4+^ is displaced from the center of a (NiO_3_)^4−^ framework to lower the number of nearest-neighbor oxygen atoms from 12. However, on removal of the pressure at room temperature and heating to only 400 K, the perovskite PbNiO_3_ transforms irreversibly to the LiNbO_3_ structure, which also consists of a framework of corner-shared octahedra. In the LiNbO_3_structure, the framework is rhombohedral (R-3); the Pb^4+^ are displaced along a [111] axis into an octahedral site. As pointed out [11] and illustrated in figure 5, this transformation only requires a rearrangement of the cooperative rotations of the corner-shared octahedra of the framework that is triggered by a different displacement of the Pb^4+^ ions. The LiNbO_3_ structure of PbNiO_3_ is more stable at ambient pressure, which is why the transformation is irreversible on thermal cycling. However, under pressure the ambient-pressure PbNiO_3_ can be converted back to the perovskite structure in which the Pb^4+^ ion has a larger oxygen coordination. The geological literature has recognized that the LiNbO_3_ structure commonly transforms to perovskite under high pressure.

### 2.8. PbRuO_3_

A large cubic-field splitting of the 4d states stabilizes the low-spin configuration *t*^*4*^*e*^*0*^ at an octahedral-site Ru^IV^. The Sr_1−*x*_Ca_*x*_RuO_3_ system can be prepared as perovskites under ambient pressure; the Sr_1-*x*_Ba_*x*_RuO_3_ perovskites with *x* > 0 have a tolerance factor *t* > 1 and require a high-pressure synthesis to form as cubic perovskites [41–43]. In ferromagnetic SrRuO_3_, the 4d electrons of the low-spin t^4^e^0^ manifold are at the crossover between localized and itinerant behavior; [42] the RuO_3_ framework of the system Sr_1−*x*_Ca_*x*_RuO_3_ evidences a Griffiths-type ferromagnetism with an eventual vanishing of long-range magnetic order in CaRuO_3_ [41]. The high-pressure Sr_1−*x*_Ba_*x*_RuO_3_ cubic phase exhibits strong itinerant-electron ferromagnetism; in BaRuO_3_, there is an abrupt loss of long-range magnetic order at a critical pressure *P*_c_ 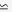
8.6 GPa [44] indicative of a first-order transition of the majority-spin electrons to itinerant behavior like the minority-spin electrons.

**Figure 6.**
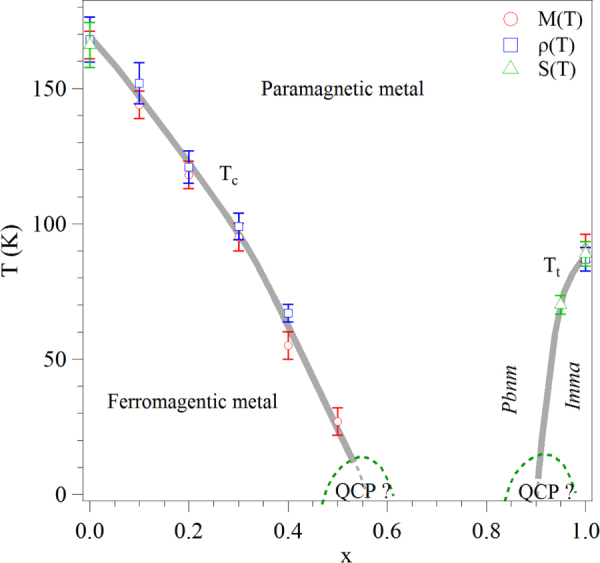
Phase diagram of Sr_1−*x*_Pb_*x*_RuO_3_; the data are from [14].

**Figure 7.**
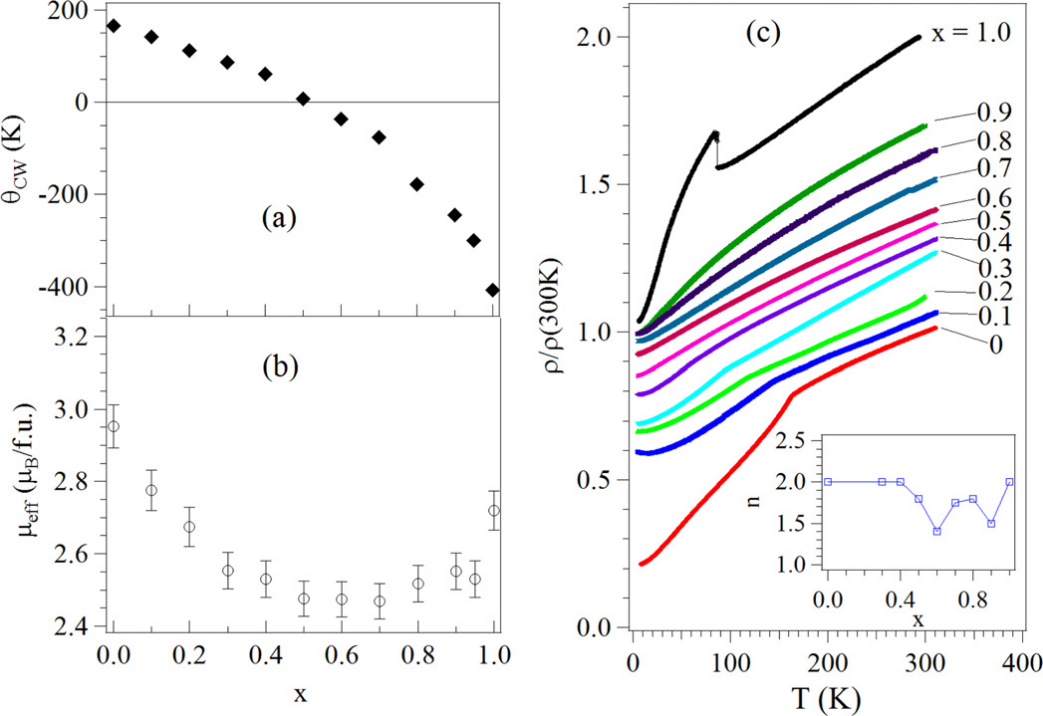
(a), (b) Parameters from fitting the paramagnetic susceptibility to a Curie–Weiss law and (c) temperature dependence of resistivity *ρ* of Sr_1−*x*_Pb_*x*_RuO_3_; the inset: *n* versus *x* obtained from the power-law analysis of *ρ*(*T*); the data are from [14].

Although PbRuO_3_ has a perovskite tolerance factor *t* ≤ 1, PbRuO_3_ crystallizes at ambient pressure in the oxygen-deficient pyrochlore structure Pb_2_Ru_2_O_7-*x*_ with 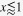 in which Pb–6s, 6p hybrids project electron density into the oxygen vacancies. However, PbRuO_3_ can be stabilized as an orthorhombic (*Pbnm*) perovskite at 9 GPa and 1400 °C;[45] but this phase transforms at ambient pressure to another orthorhombic (*Imma*) perovskite phase below 90 K [12]. Since cracks are created in the sample during the first-order transition from *Pnma* to the *Imma* phase, the *Imma* phase was initially reported to be an insulator [12]. By synthesizing the PbRuO_3_ perovskite phase in a Walker-type multianvil module, Cheng *et al* [13, 14] were able to obtain a sample with large enough grains to measure the transport properties on a single grain; they showed that the perovskite PbRuO_3_ becomes a metal in the *Imma* phase as a result of broadening of the *π*∗ band and that the phase change below 90 K under ambient pressure is not caused by changes in the perovskite tolerance factor, but by an interaction between the Pb–6s, 6p hybrids and the Ru*–*4d electrons that broadens the *π*∗ band. In the perovskite system Sr_1−*x*_Pb_*x*_RuO_3_, this interaction broadens the Ru–4d band to suppress systematically with increasing *x* the ferromagnetism of SrRuO_3_, figure 6 [14]. Plots of the Weiss constant *θ*_CW_ and the *μ*_eff_ obtained from the paramagnetic susceptibility, figures 7(a) and (b), show that suppression of the long-range magnetic order occurs where *θ*_CW_ passes through zero. A power-law analysis of the low-temperature resistivity *ρ* (*T*) = *ρ*_o_ + aT^n^ to obtain the *x* dependence of *n* is shown in figure 7(c); the minimum *n* at *x* = 0.6 indicates that suppression of the magnetism is associated with the existence of a quantum critical point (QCP); the second minimum of *n*(*x*) at *x* 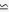 0.9 is a QCP associated with the appearance of the low-temperature *Imma* phase of perovskite PbRuO_3_. The low-temperature *Imma* phase can be suppressed by applying hydrostatic pressure [13, 46]. Kusmartseva *et al* [46] have monitored the transition by measuring resistivity *ρ*(*T*) under different pressures. They have found that the phase transition temperature *T*_t_ lowers gradually as pressure increases; it vanishes at about 5 GPa. The power-law analysis of *ρ*(*T*) under different pressures also confirms a QCP at around 5 GPa.

**Figure 8.**
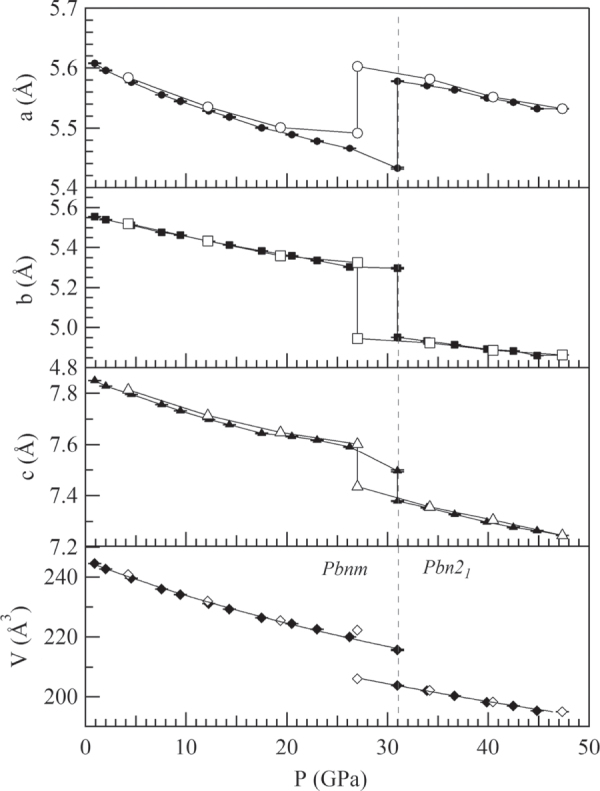
Lattice parameters as a function of pressure for PbRuO_3_; the data are from [48].

Application of hydrostatic pressure *P* at room temperature to the PbRuO_3_ orthorhombic (*Pbnm*) perovskite phase suppresses the transition to the metallic *Imma* phase at low temperature. More interesting, the room-temperature lattice parameters and volume *V* of the *Pbnm* phase show a systematic contraction with *P* (figure 8) until, at a *P*_c_ ≈ 32 GPa,the lattice parameter *a* jumps and the lattice parameters *b*, *c* and volume *V* drop abruptly at a first-order transition to a lower-symmetry *Pbn*2_1_ phase. The volume decrease is about 6%. This behavior contrasts with that of a normal *Pbnm* perovskite, which transforms under pressure to a higher symmetry R-3c phase [47].

**Figure 9.**
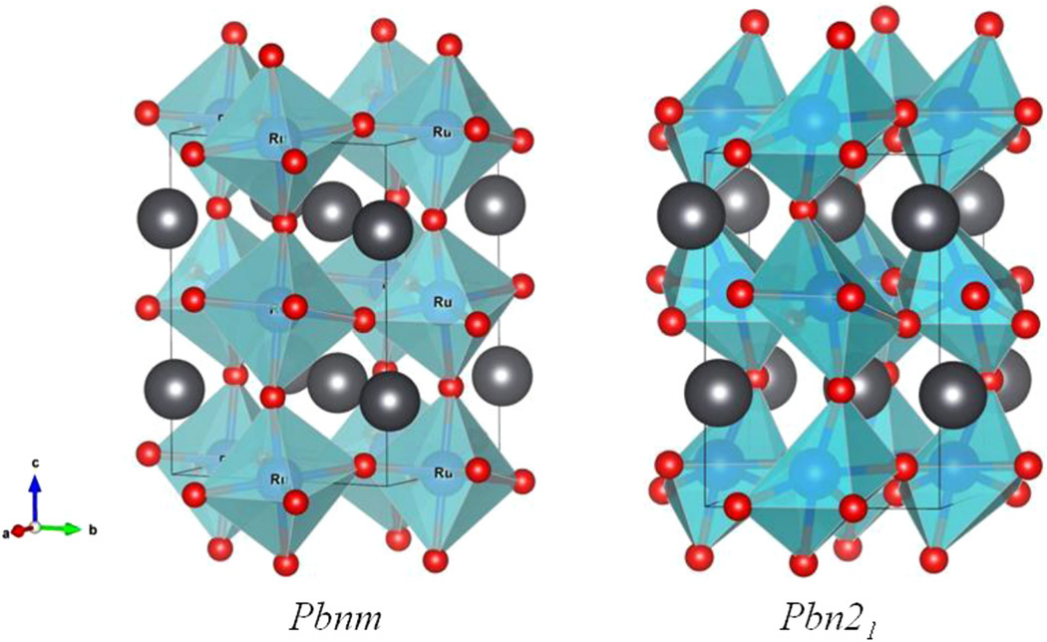
Two structural models of PbRuO_3_.

The PbRuO_3_ perovskite has an ambient-pressure tolerance factor *t* < 1, and a cooperative rotation of the RuO_6/2_ octahedra about a cubic [1-1 0] axis (*b*-axis of orthorhombic *Pbnm*) shifts the Pb atoms from the A-site center to coordinate it with fewer, but closer oxygen-atom nearest-neighbors. The small shift of the Pb atoms results in four Pb–Ru distances across a shared site face about 0.05 Å shorter than the average value. A distortion of the octahedral sites accompanies the cooperative rotation of the octahedra. In the *Pbnm* structure with a tolerance factor *t* near unity, the principal site distortion is a reduction from 90° of the O_2_–Ru–O_2_ bond angle that subtends the rotation axis [14]. In contrast, the cooperative rotation of highly distorted RuO_6/2_ octahedra in the *Pbn*2_1_ phase of figure 9 creates eight different Pb–Ru distances [48]. Most striking is a single Pb–Ru distance as short as 2.6 Å, about 0.6 Å shorter than the average value, which is reduced only slightly on traversing *P*_c_. Kusmartseva *et al* [46] have also found independently the pressure-induced phase transition at *P* ≈ 30 GPa by their structural study under high pressure. They have given only the change of lattice parameters on crossing the phase boundary.

**Figure 10.**
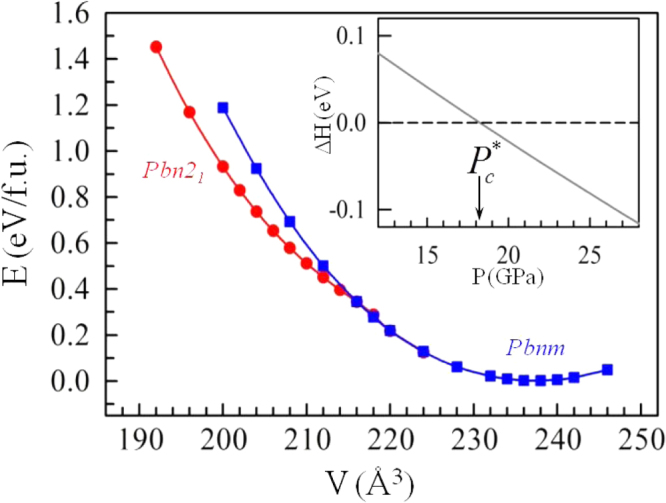
Variations in the relative internal energies (*E*) per formula unit (f.u.). for the *Pbnm* and *Pbn*2_1_ phases of PbRuO_3_ as a function of volume (*V*) from the LDA calculation. Inset shows the enthalpy difference between the *Pbnm* and *Pbn*2_1_ phases (Δ*H* = *H*_*Pbn*2_1__ − *H*_*Pbnm*_) as a function of pressure (*P*). The data are from [48].

A DFT calculation has shown [48] that the short Pb–Ru bond is the result of an interaction of the Pb^2+^ lone-pair electrons with the two holes per Ru in the 4d*-*electron *π*∗ band in a shared-electron reaction Pb^2+^ + Ru^IV^ = Pb^4+^ + Ru^II^ shifted to the left. Figure 10 shows the variations with volume in the relative internal energies per formula unit as obtained by the DFT calculation. By fitting the *E*–*V* data to the third-order Birch–Murnaghan equation, the enthalpy difference Δ*H* between the two phases (figure 10 inset) clearly demonstrates the phase crossover. A density of states plot of the *Pbnm* phase at ambient pressure shows an overlap of the Pb–6s, Ru–4d, and O–2p states in the energy range *E*_*F*_−3 eV < *E* < *E*_*F*_ + 1 eV. The covalent-like Pb–Ru bond formation in the *Pbn*2_1_ phase leaves the corresponding antibonding orbitals completely empty; *E*_*F*_ is located at the leading edge of the bonding states, which accounts for the observation that the *Pbn*2_1_ phase remains a bad metal.

## 3. Conclusions

This review of the observed properties of the Pb*M*O_3_ perovskites with *M* = Ti, Cr, Mn, Fe, Ni, and Ru leads to the following conclusions.

(1) A perovskite tolerance factor *t* > 1 for Pb*M*O_3_ with *M* = Ti, Cr, Mn, Fe, Ni makes it necessary to use high-pressure synthesis to obtain a cubic 3C perovskite phase. Although PbRuO_3_ has a *t* < 1, the Pb^2+^ lone pair stabilizes an oxygen-deficient pyrochlore, so here also high-pressure synthesis is needed to obtain a perovskite phase.
(2) Where the cubic phase is stable at ambient temperature and pressure, a *t* > 1 stretches the Pb–O bonds from their equilibrium length to make the phase unstable with respect to a Pb^2+^ displacement from the center of symmetry of its site with an associated hybridization of the Pb–6s,6p orbitals, but creation of a polar state is confined by symmetry to a transition from a cubic to a tetragonal phase. In PbTiO_3_ and PbVO_3_ where the M^IV^ cation has a 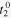 or a 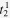 electron configuration, a *t* > 1 also stretches the equilibrium O–*M*–O bonds to provide cooperative Pb^2+^ and M^IV^ ferroic displacements that transition the lattice from cubic to tetragonal symmetry. The 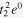 configuration on octahedral-site Cr^IV^ prohibits a cooperative ferroic displacement of the Cr^IV^ in a cubic-to-tetragonal transition. In this case, the Cr^IV^ electrons force a non-colinear displacement of the Pb^2+^ ions that is cooperative with a charge disproportionation, 3Cr^IV^ = 2Cr^III^ + Cr^VI^, on the CrO_3_ sublattice.
(3) As the atomic number of the *M* atoms increases, the energies of the M^IV^/M^III^ and M^III^/M^II^ redox energies decrease; where they cross the top of the Pb−6s band, electron transfer from the Pb^2+^−6s to the M^IV^/M^III^ or even to the M^III^/M^II^ redox couple occurs. This electron transfer reduces the perovskite tolerance factor to a *t* < 1 and an orthorhombic structure in which the Pb-atom displacements are non-ferroic.
(4) With three half-filled *π*-bonding d orbitals on M^IV^:d^3^ ions in octahedral sites, ferroic M^IV^ displacements, charge disproportionation, and orbital ordering cannot occur. Moreover, Pb^2+^ displacements along a tetragonal axis are opposed by *M*–O–*M* interactions, which limits a displacement of the Pb^2+^ ion as has been argued by Hill [49].
(5) Competition between the Pb^2+^−6p and the empty M^IV^−3d orbitals for covalent bonding with the O−2p_*π*_ orbitals of the *M*O_3_ sublattice narrows any *π*∗ band in a single-valent *M*O_3_ perovskite sublattice; this competition may induce a transition from itinerant to localized d-electron behavior. Where the M^IV^:d^2^ electrons are localized by the band narrowing, applying hydrostatic pressure can induce a first-order transition to itinerant d electrons with a Mott–Hubbard *U* > *W*; and applying additional pressure may give a smooth transition to metallic conduction with a *W* > *U*.
